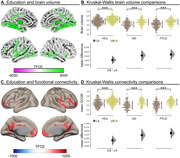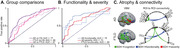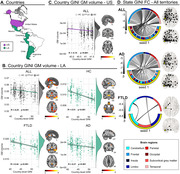# Multilevel measures of socioeconomic disparities impact brain health and dementia across the Americas

**DOI:** 10.1002/alz.088158

**Published:** 2025-01-09

**Authors:** Agustina Legaz, Raul Gonzalez‐Gomez, Joaquín Migeot, Stefanie Danielle Pina Escudero, Florencia Altschuler, Agustin Ibañez

**Affiliations:** ^1^ Cognitive Neuroscience Center (CNC), Universidad de San Andres, Buenos Aires Argentina; ^2^ Latin American Brain Health Institute (BrainLat), Universidad Adolfo Ibañez, Santiago Chile; ^3^ Global Brain Health Institute (GBHI), University of California, San Francisco USA; ^4^ Global Brain Health Institute (GBHI), Trinity College Dublin (TCD), Dublin Ireland

## Abstract

**Background:**

Socioeconomic disparities (SED) influence brain health and dementia. Latin America (LA) is characterized by high SED and a disproportionate prevalence of Alzheimer’s disease (AD) and frontotemporal lobe degeneration (FTLD) compared to high‐income populations like the United States (US). However, the impact of SED on brain reserve across neurocognitive pathways related to aging and dementia in LA remains unknown.

**Method:**

We evaluated how SED impact brain volume and functional connectivity in participants with AD, FTLD, and controls from LA (Argentina, Chile, Colombia, Mexico, Peru) and US, triangulating three SED measures: (i) educational attainment (*n* = 1410), (ii) cross‐culturally harmonized social determinants of health (SDH; *n* = 2324), and (iii) structural inequality (GINI index; *n* = 2135). Data were obtained from the Multi‐Partner Consortium to Expand Dementia Research in Latin America [ReDLat], the Alzheimer’s Disease Neuroimaging Initiative [ADNI], and the Laboratory of Neuro Imaging [LONI]. We controlled for multiple potential confounders (e.g., scanner, TIV, age, sex).

**Result:**

Across the three studies, SED was associated with impaired neurocognitive outcomes, especially in LA compared to the US. (i) Lower educational attainment was associated with reduced fronto‐temporo‐posterior brain volume and connectivity across all LA groups (**Fig 1**; multiple regressions and kruskall‐wallis comparisons, *P*<0.05 TFCE correction). (ii) Adverse SDH including childhood SDH, discriminated between controls, AD, and FTLD in LA, affecting patients’ cognition, severity, and functionality (**Fig 2A‐B**; XGBoost binary logistic regression). Also, adverse SDH induced reduced volume and connectivity (VBM and whole‐brain analysis, both *P*‐FDR<0.05) across LA groups (**Fig 2C**). Finally, (iii) Higher structural income inequality at both the state/country levels was associated with reduced volume and connectivity in the temporo‐parietal, cingulum‐limbic, and cerebellar regions, with more pronounced effects in LA groups (**Fig 3**; multiple regressions with grey matter volume [w‐maps, *P*‐FWE<0.05] and connectivity [wSDM coefficients, *P*‐Bonf<0.05]).

**Conclusion:**

Findings suggest that exacerbated SED, observed in more diverse samples from LA, differentially and negatively impact neurocognitive factors in aging and dementia. This aligns with the notion that SED diminish brain and cognitive reserve, amplifying dementia burden. Tailored, local‐sensitive models are required to leverage the impact of SED in brain health and dementia, particularly in underrepresented populations like LA.